# Co-expression of nuclear and cytoplasmic HMGB1 is inversely associated with infiltration of CD45RO+ T cells and prognosis in patients with stage IIIB colon cancer

**DOI:** 10.1186/1471-2407-10-496

**Published:** 2010-09-16

**Authors:** Rui-Qing Peng, Xiao-Jun Wu, Ya Ding, Chun-Yan Li, Xing-Juan Yu, Xing Zhang, Zhi-Zhong Pan, De-Sen Wan, Li-Ming Zheng, Yi-Xin Zeng, Xiao-Shi Zhang

**Affiliations:** 1 State Key Laboratory of Oncology in South China, 651 Dongfeng R E, 510060, Guangzhou, China; 2 Biotherapy Center, Cancer Center, Sun Yat-sen University, 651 Dongfeng R E, 510060, Guangzhou, China; 3 Department of Colorectal Surgery, Cancer Center, Sun Yat-sen University, 651 Dongfeng R E, 510060, Guangzhou, China; 4 Department of Experimental Research, Cancer Center, Sun Yat-sen University, 651 Dongfeng R E, 510060, Guangzhou, China; 5 Biotherapy Center, The First Affiliated Hospital, Chongqing Medical University, 1 Youyi R, 400016, Chongqing, China

## Abstract

**Background:**

The intratumoral infiltration of T cells, especially memory T cells, is associated with a favorable prognosis in early colorectal cancers. However, the mechanism underlying this process remains elusive. This study examined whether high-mobility group box 1 (HMGB1), a damage-associated molecular pattern (DAMP) molecule, is involved in the infiltration of T cells and disease progression in locally advanced colon cancer.

**Methods:**

Seventy-two cases of pathologically-confirmed specimens were obtained from patients with stage IIIB (T3N1M0) colon cancer who underwent radical resection between January 1999 and May 2002 at the Cancer Center of Sun Yat-Sen University. The density of tumor-infiltrating lymphocytes (TILs) within the tumor tissue and the expression of HMGB1 in the cancer cells were examined via immunohistochemical analysis. The phenotype of CD45RO+ cells was confirmed using a flow cytometric assay. The association between HMGB1 expression, the density of TILs, and the 5-year survival rate were analyzed.

**Results:**

The density of CD45RO+ T cells within the tumor was independently prognostic, although a higher density of CD3+ T cells was also associated with a favorable prognosis. More importantly, the expression of HMGB1 was observed in both the nucleus and the cytoplasm (co-expression pattern) in a subset of colon cancer tissues, whereas nuclear-only expression of HMGB1 (nuclear expression pattern) existed in most of the cancer tissues and normal mucosa. The co-expression pattern of HMGB1 in colon cancer cells was inversely associated with the infiltration of both CD3+ and CD45RO+ T cells and 5-year survival rates.

**Conclusions:**

This study revealed that the co-expression of HMGB1 is inversely associated with the infiltration of CD45RO+ T cells and prognosis in patients with stage IIIB colon cancer, indicating that the distribution patterns of HMGB1 might contribute to the progression of colon cancer via modulation of the local immune response.

## Background

Colorectal cancer is one of the most common causes of cancer deaths worldwide [1]. The median overall survival of patients with metastatic colorectal cancer has increased from 12 months to approximately 24 months over the past decade as a result of improvements in systemic therapies, including new chemotherapeutic agents such as irinotecan and oxaliplatin and monoclonal antibodies against epidermal growth factor receptor (EGFR) and vascular endothelial growth factor (VEGF). However, the 5-year survival of patients is still poor [2-5]. Thus, one of the main challenges for the treatment of colorectal cancer remains to identify new strategies beyond chemotherapy to inhibit disease progression.

Colorectal cancers are characterized by infiltration with multiple stromal cells, among which are tumor-infiltrating lymphocytes (TILs) that act as prognostic and predictive factors [6-15]. TILs include natural killer (NK) cells, CD8+ T cells, and CD4+ T cells, including Th1, Th2, Th17, and Treg cells. Although the role of TILs in tumor progression is controversial, CD45RO+ T cells have been identified as the main anti-tumoral effectors in early colorectal cancers. Hierarchical clustering has shown that markers of T-cell migration, activation, and differentiation are increased in tumors without signs of early metastatic invasion. These tumors have an increased number of CD8+ T cells, ranging from early memory (CD45RO+, CCR7-, CD28+, and CD27+) to effector memory (CD45RO+, CCR7-, CD28-, and CD27-) T cells. The presence of high levels of infiltrating memory CD45RO+ T cells is correlated with the absence of signs of early metastatic invasion, a less advanced pathologic stage, and increased survival, which has been confirmed in several series of patients [16,17]. These correlations indicates that protective immune responses exist in a subset of colorectal cancer patients. Activated tissue-resident memory T cells, which have a potent lytic potential with the expression of perforin and granzyme B, are not only capable of providing immediate effector function at the site of cancer cells but can also generate an effective secondary immune response [18-21]. Radiotherapy and some chemotherapeutics have the potential to induce the immunogenic death of cancer cells and subsequently activate memory T cells. Furthermore, clinical trials have shown that the efficacy of chemotherapy against colon cancer can be improved when combined with cytokines [22-27]. These data indicate that the activation of memory T cells that have infiltrated into tumor tissues might be a promising therapeutic strategy.

Tissue-resident memory T cells result from the acute immune response, which may be induced by damage-associated molecular pattern molecules (DAMPs). High-mobility group box 1 (HMGB1), previously named HMG1, amphoterin, and sulfoglucuronyl carbohydrate binding proteinis a DAMP. Under physiologic conditions, HMGB1 localizes in the nucleus. HMGB1 is released into the extracellular matrix by spontaneous release or by dying cells. HMGB1 is a multi-functional protein that accelerates cell growth, invasion, and angiogenesis in cancer tissues, induces apoptosis in macrophages, and promotes immune responses [28-31]. With respect to modulation of the immune response, HMGB1 binds to surface receptors expressed on dendritic cells (DCs), leading to the maturation of DCs, antigen processing, and a subsequent protective effect. There is also evidence that HMGB1 induces immune tolerance by interfering with the antigen-presenting process and decreasing the number of DCs or by binding to the TLR9 agonist, which leads to human plasmacytoid DC suppression [32-36]. In addition, extracellular HMGB1 triggers the inflammatory cascade through multiple pathways, facilitating progression of the tumor.

Because HMGB1 has a dual effect on tumor progression, the feasibility of targeting the release of HMGB1 remains elusive. To this end, this study determined whether the expression of HMGB1 is related to the infiltration of T cells and patient prognosis in locally advanced colon cancers.

## Methods

### Materials

Seventy-two cases of pathologically-confirmed specimens matched with adjacent normal mucosa were obtained from patients with stage IIIB (T3N1M0; AJCC, 2002) colon cancer between January 1999 and May 2002 at the Cancer Center of Sun Yat-Sen University in Guangzhou, China (Table 1). All of the patients underwent radical resection and 5-FU-based adjuvant chemotherapy post-operatively for 6 months. Patients were evaluated every 3 months during the 1^st ^year, every 6 months in the 2^nd ^year, and by telephone or mail communication once every year thereafter for a total of 5 years. If recurrence or metastasis occurred, 5-FU-based chemotherapy was given according to the NCCN guidelines. Overall survival was defined as the time from surgery to death. Alternatively, censoring was done at the last known date the patient was alive. This study was approved by the institutional ethical review committee of Sun Yat-Sen University Cancer Center.

**Table 1:** Patient Characteristics (N = 72)

Characteristic	No. of patients (%)
**Age, years**	
< 60	33(45.8)
≥60	39(54.2)
**Gender**	
Male	40(55.6)
Female	32(44.4)
**Tumor sites**	
Left hemicolon	46(63.9)
Right hemicolon	26(36.1)
**Pathological grade**	
G1	10(13.9)
G2	54(75.0)
G3	8(11.1)
**Survival time, months**	
≥60	52(72.2)
< 60	20(27.8)

### Immunohistochemical assay and scoring systems

Formalin-fixed, paraffin-embedded archived tissues were cut into 4-μm sections. The size of each tissue section was about 1.0 cm×1.5 cm. Then, the sections were de-waxed, rehydrated, blocked with hydrogen peroxide, and the antigens were retrieved in a microwave in 10 mM citrate buffer (pH 6.0) for 10 minutes and cooled to room temperature. After blocking with sheep serum, the sections were incubated overnight at 4°C with either rabbit polyclonal antibody against human HMGB1 at a dilution of 1:1000 (Abcam, Cambridge, MA, USA) or mouse monoclonal antibody against human CD3, CD45RO, CD4, CD8, and CD56. All of these antibodies (Zymed, San Diego, CA, USA) were diluted 1:100. Subsequently, biotinylated secondary antibodies and streptavidin-biotinylated horseradish peroxidase complex were used. The sections were developed with diaminobenzidine tetrahydrochloride (DAB) and counterstained with hematoxylin. Negative controls were employed in which the primary antibody was replaced by phosphate-buffered solution (PBS).

The density of TILs within the tumors and the expression of HMGB1 in cancer cells were scored with two scoring systems. Hussein's method was used to score the density of TILs as follows: 1) the cells were counted in at least 10 different fields of each section, and the size of each high-power field (×400) was about 300 μm×300 μm; 2) the cells were counted in the tumor stroma; 3) the areas of highest density were chosen; 4) necrotic areas were avoided; 5) two observers counted the cells at the same time and in the same field using a multiple-lens microscope; and 6) the results were expressed as the mean ± standard error of the mean [37]. The expression of HMGB1 was interpreted via immunoreactivity using the 0-4 semi-quantitative system derived from Soumaoro [38] for both the intensity of staining and the percentage of positive cells (labeling frequency percentage). The intensity of nuclear or cytoplasmic staining was grouped into the following four categories: no staining/background of negative controls (score = 0), weak staining detectable above background (score = 1), moderate staining (score = 2), and intense staining (score = 3). The labeling frequency was scored as 0 (≤1%), 1 (1%-24%), 2 (25%-49%), 3 (50%-74%), and 4 (≥75%). The sum index was obtained by totaling the intensity and percentage scores, as follows: (-), (+), (++), and (+++) indicated sum-indexes of 0-1, 2-3, 4-5, and 6-7, respectively; (-) and (+) were defined as no or modest expression, and (++) and (+++) were defined as strong expression. Each section was independently scored by two pathologists. If an inconsistency occurred, a third pathologist was consulted to achieve consensus.

### Double immunohistochemistry for HMGB1 and CD45RO

Double immunohistochemical staining was performed with a Polymer kit (GBI, Seattle, WA, USA). The sections were simultaneously incubated with two primary antibodies derived from different species. After blocking nonspecific binding with goat serum, the sections were incubated overnight at 4°C with a rabbit anti-human HMGB1 polyclonal antibody (1:1000; Abcam, Cambridge, MA, USA) and a mouse anti-human CD45RO monoclonal antibody (1:100; Zymed, San Diego, CA, USA) simultaneously. After washing in PBS, the sections were incubated with both alkaline phosphatase-labeled goat anti-rabbit IgG antibodies and horse radish peroxidase-labeled goat anti-mouse IgG antibodies with a definite percentage for 30 minutes at room temperature. After three washes with PBS, sections were developed sequentially with DAB and AP-Red and counterstained with hematoxylin. Negative controls were employed in which both of the primary antibodies were replaced with PBS.

### Flow-cytometric analysis

Fresh tumor specimens were processed via sterile mechanical dissection. The tissue was cut into small pieces and stirred for 2 hours at 37°C in an enzymatic bath containing RPMI 1640 (Invitrogen, San Diego, CA, USA) and HEPES buffer (20 mmol/L) containing penicillin/streptomycin (120 μg/ml and 100 ug/ml, respectively), fungizone (0.25 mg/ml), ceftazidime (50 μg/ml), collagenase type III (200 U/ml), and DNase type I (100 U/ml). The suspension was then filtered through two wire grids (70 and 40 μm), and the cells were washed three times with Hank's balanced salt solution (HBSS). Subsequently, cells were separated on a Percoll density gradient (Pharmacia Biotech AB, Uppsala, Sweden) for 30 min at 1500 × *g *at room temperature. The dense layer, enriched for lymphocytes, was collected and washed. Then the cells were incubated for 30 minutes at 4°C with antibodies conjugated to APC against CD3 (Biolegend, San Diego, CA, USA) and phycoerythrin (PE) against CD45RO (Biolegend, San Diego, CA, USA). Analyses were performed with a five-color fluorescence-activated cell sorter (FC500; Beckman-Coulter, Fullerton, CA, USA) and Cxp cytometer software (Beckman-Coulter, Fullerton, CA, USA). Immune subpopulations were measured as a percentage of the total number of all cells and a percentage of the total number of CD3+ cells.

### Statistical analysis

The correlation between the density of TILs or HMGB1 expression with patient characteristics and the correlation between the density of TILs and levels or subcellular location of HMGB1 were analyzed with a chi-square test or Fisher's exact test. The following factors were assessed with both univariate and multivariate analyses to determine their influence on overall survival: gender, age, pathologic grade, tumor site, the density of CD3+ cells, the density of CD45RO+ cells, and the level or subcellular location of HMGB1 expression within colon cancer tissues. Kaplan-Meier curves were used to estimate the distributions of those variables in relation to survival, which were compared using the log-rank test. The Cox regression model was used to correlate assigned variables with overall survival. All statistical analyses were carried out using SPSS 13.0 software (SPSS Inc., Chicago, IL, USA). Statistical significance was assumed for a two-tailed *P *< 0.05.

## Results

### Patterns of TIL infiltration and HMGB1 expression within stage IIIB colon cancer tissues

**Figure 1:**
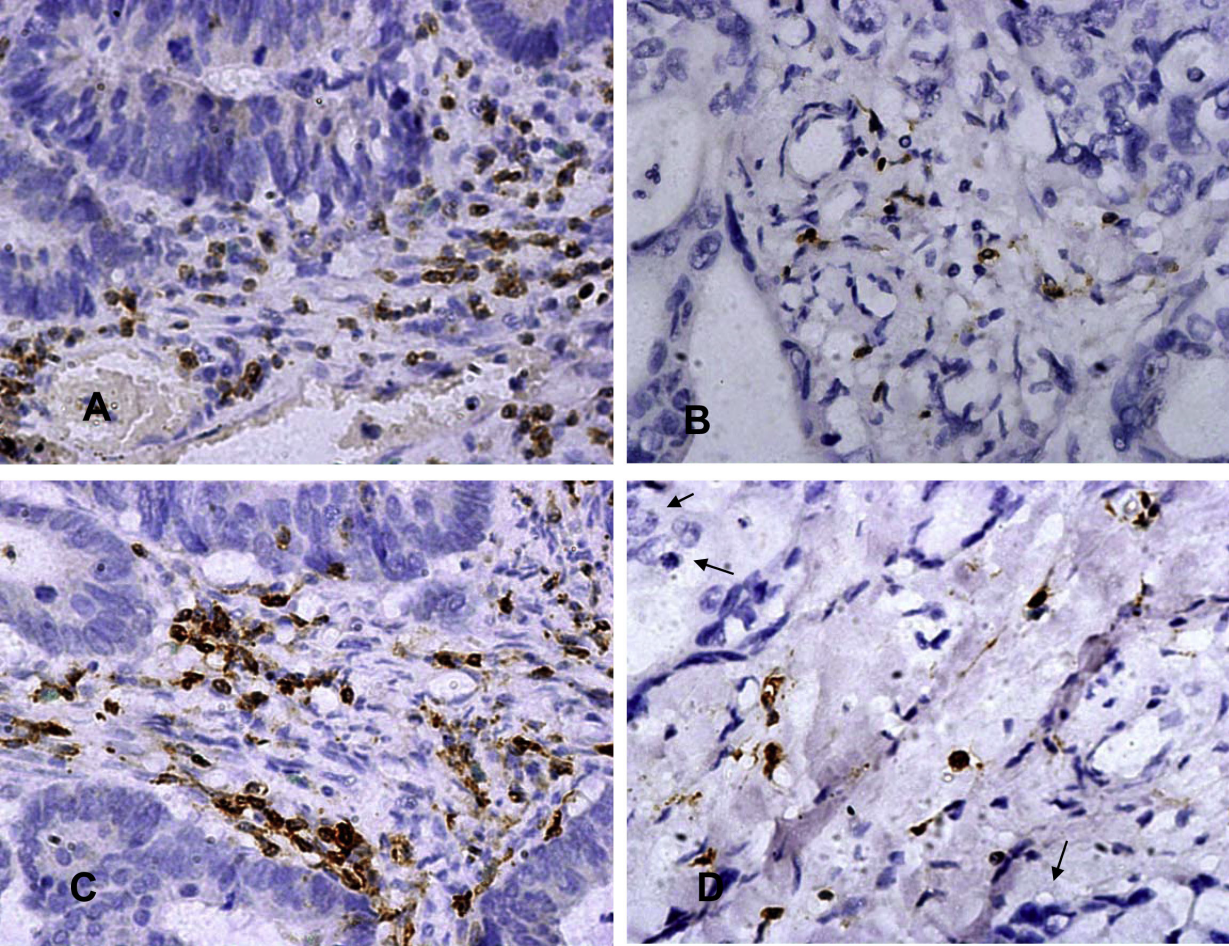
**Patterns of TILs within stage IIIB colon cancer tissues**. A high density of CD3+ cells infiltrated into the colon cancer stroma (×400, A), a low density of CD3+ cells infiltrated into the colon cancer stroma (×400, B), a high density of CD45RO+ cells infiltrated into the colon cancer stroma (×400, C), and a low density of CD45RO+ cells infiltrated into the colon cancer stroma (×400, D, "↑"refers to tumor tissue). All of the antigens were immunolocalized to the membrane.

CD3+ and CD45RO+ cells were observed in all cases in the tumor stroma and the adjacent normal mucosa to different extents. Both antigens stained the cell membrane. In addition, we performed immunostaining against CD4, CD8, and CD56 in 10 of the tissue sections to determine which of these proteins might play an important role in this group of patients. Among the CD3+ cells, CD8+ cells outnumbered CD4+ cells, while CD56+ cells were rare (Fig. 1, 2. To confirm the phenotype of the CD45RO+ cells, co-expression of CD3 with CD45RO was examined in five fresh cancer tissues via flow cytometry, which indicated that 72% of all tumor-infiltrating CD45RO+ lymphocytes were also CD3-positive (Fig. 3).

**Figure 2:**
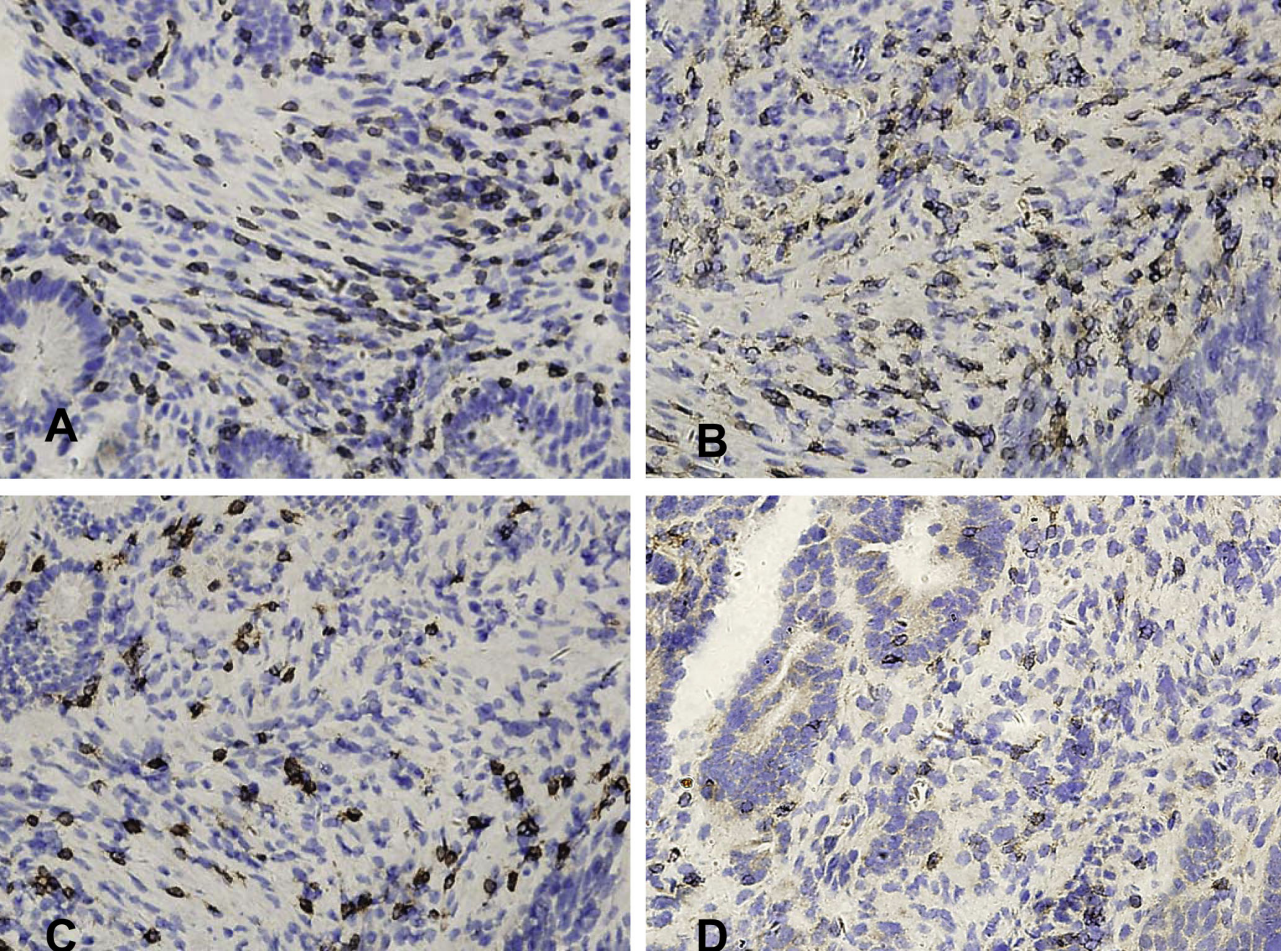
**Subtype of CD3+ cells within stage IIIB colon cancer tissues**. A high density of CD3+ cells infiltrated into the colon cancer stroma (×400, A); CD4+ cells infiltrated into the colon cancer stroma (×400, B), CD8+ cells infiltrated into the colon cancer stroma (×400, C), and CD56+ cells infiltrated into the colon cancer stroma (×400, D). All of the antigens were immunolocalized to the membrane.

**Figure 3:**
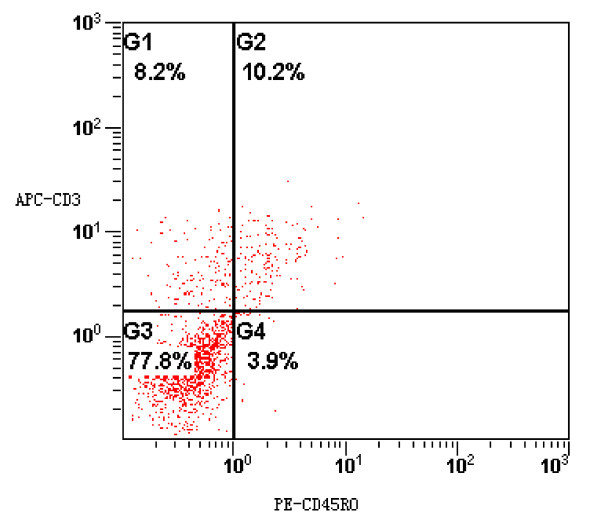
**Co-expression of CD3 and CD45RO antigens among TILs analyzed with flow cytometry**.

**Figure 4:**
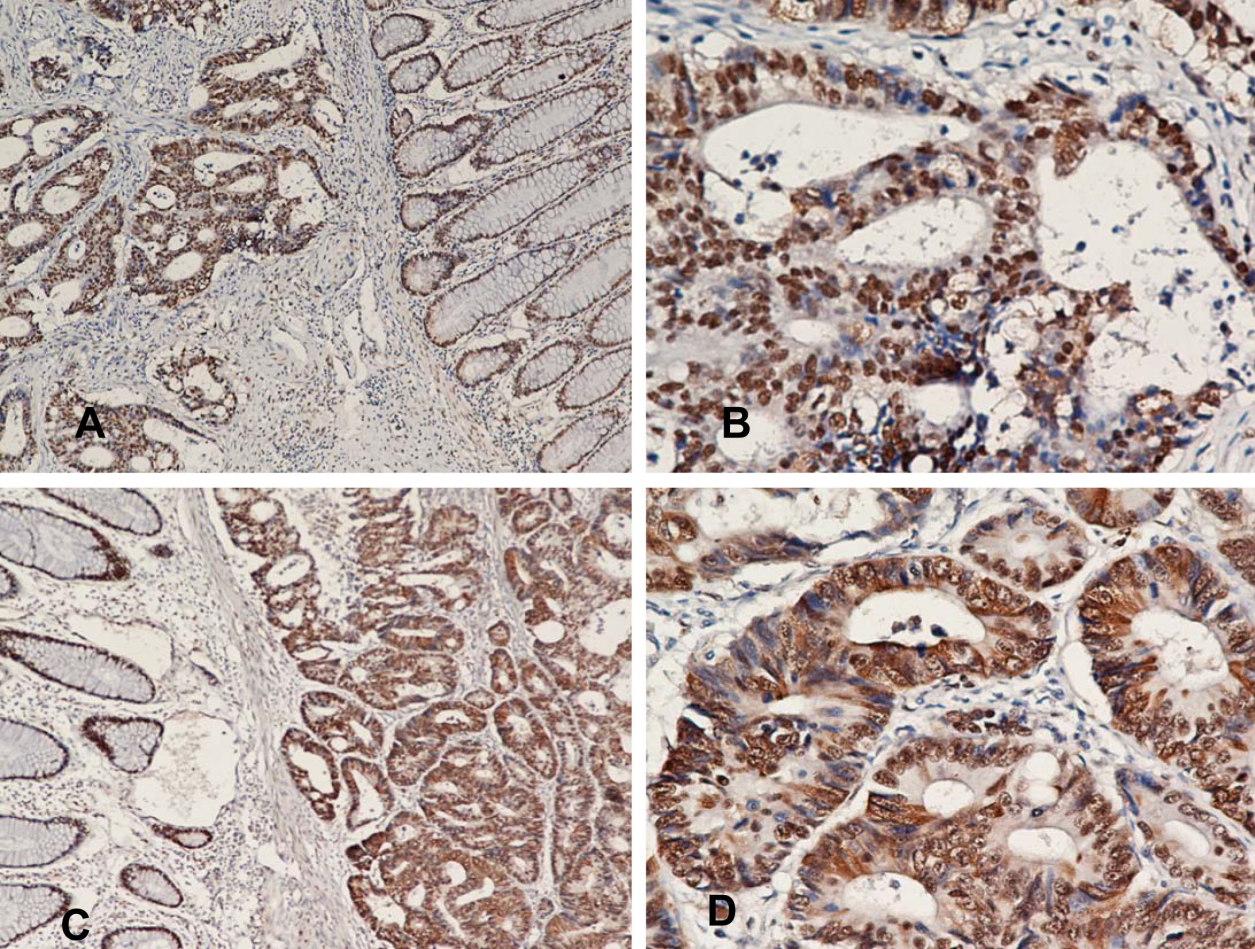
**Expression of HMGB1 in colon cancer and the adjacent normal mucosa**. Nuclear staining of HMGB1 (×100 in A and ×400 in B) and nuclear and cytoplasmic staining of HMGB1 (×100 in C and ×400 in D).

Although HMGB1 staining existed only in the nucleus in normal mucosa, co-expression of nuclear and cytoplasmic HMGB1 (co-expression pattern) existed in a subset of cancer tissues (12/65 [18.5%]), whereas most of the cancer tissues only showed nuclear staining of HMGB1 (nuclear expression pattern; 53/65 [81.5%]; Table 2, Fig. 4).

**Table 2:** Patterns of HMGB1 expression in stage IIIB colon cancers (N = 72)

	**HMGB1 expression**			
**0**	**+**		**++-+++**	
		
	nuclear only	nuclear and cytoplasmic	nuclear only	nuclear only nuclear and cytoplasmic
7	20	5	33	7

### The relationship between HMGB1 expression and the density of CD3+ or CD45RO+ cells

**Figure 5:**
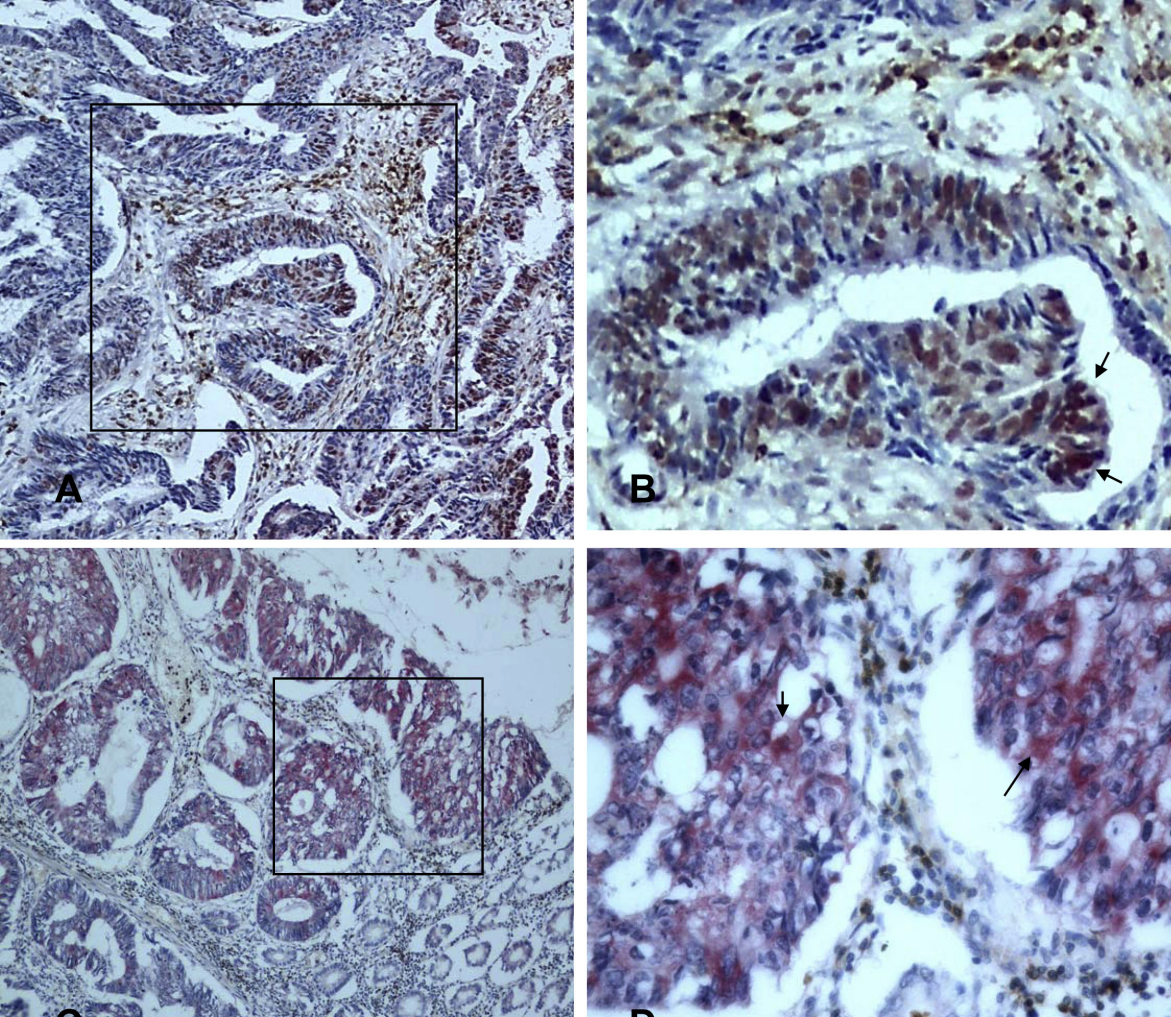
**Double staining of HMGB1 and CD45RO+ cells in stage IIIB colon cancer (red staining of HMGB1 in cancer cells and brown staining of CD45RO+ cells in the stroma)**. Strong nuclear staining of HMGB1 was associated with a high density of CD45RO+ cells infiltrating into colon cancer tissues (×100 in A and ×400 in B, "↑"refers to nuclear staining in cancer cells). Strong nuclear and cytoplasmic staining of HMGB1 was also associated with a low density of CD45RO+ cells infiltrating into colon cancer tissues (×100 in C and ×400 in D, "↑"refers to nuclear or cytoplasmic staining in cancer cells).

The density of TILs within the tumors was within the 75^th ^percentile for all those specimens in which the survival difference between the "high" and "low" groups was the largest [17]. The cut-off values for the density of CD3+ and CD45RO+ cells were 16 and 24 cells per high-power field in the center of the tumor, respectively. Thus, the density of CD3+ cells was recorded as high if > 16 cells were observed per high-power field, and the same cutoff used for CD45RO+ cells. The log-rank test was used to compare the level of HMGB1 with the density of CD3+ and CD45RO+ cells within the tumor. A stronger expression (++-+++) of HMGB1 was associated with a higher density of both CD3+ and CD45RO+ cells (Table 3, Fig. 5). Furthermore, the log-rank test was used to compare the subcellular localization of HMGB1 with the density of CD3+ and CD45RO+ cells within the tumor. The co-expression pattern of HMGB1 in the nucleus and cytoplasm was inversely associated with infiltration of both CD3+ and CD45RO+ cells, respectively (Table 4, Fig. 5).

**Table 3:** Relationship between the expression of HMGB1 and the density of TILs as well as patient characteristics (N = 72)

Characteristic	HMGB1 expression (-)- (+) (++)-(+++)		*P*
**Gender**			0.058
Male	22	18	
Female	10	22	
**Age, years**			0.113
≥60	14	25	
<60	18	15	
**Tumor sites**			0.476
Left hemicolon	19	27	
Right hemicolon	13	13	
**Pathological grade**			0.182
G1	4	6	
G2	22	32	
G3	6	2	
**Density of CD3+cells (cells per high-power field)**			0.013
≥16			
<16	20	35	0.006
**Density of CD45RO+cells (cells per high-power field)**	12	5	
≥24	19	35	
<24	13	5	

**Table 4:** Relationship between subcellular localization of HMGB1 and density of TILs (N = 65)

Subcellular localization of HMGB1			*P *value
	nuclear only	nuclear and cytoplasmic	
Density of CD3+cells (cells per high-power field)			
≥16	47	5	0.000
<16	6	7	
Density of CD45RO+cells (cells per high-power field)			
≥24	46	5	0.0001
< 24	7	7	

### Relationship between the density of TILs, HMGB1, clinical characteristics, and 5-year survival rate as analyzed via univariate analysis

The log-rank test was used to compare the density of CD3+ cells and CD45RO+ cells and the level of HMGB1 with patient characteristics. The density of CD3+ cells within the tumor stroma was associated with pathologic grades (*P*=0.023), i.e., a lower pathologic grade was associated with less CD3+ cell infiltration. However, neither the density of the CD45RO+ cells nor the levels of HMGB1 were associated with any of the clinical characteristics.

**Figure 6:**
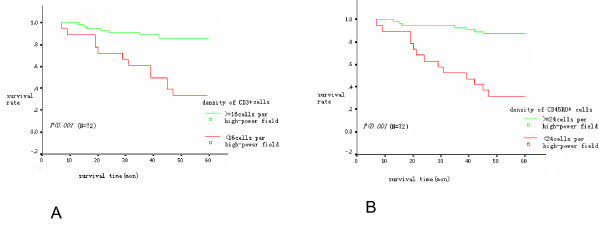
**The association between overall survival with the density of CD3+ and CD45RO+ cells as well as HMGB1 expression in stage IIIB colon cancers**. A high density of CD3+ cells within the tumor was associated with a longer 5-year survival rate (A); a high density of CD45RO+cells within the tumor was associated with a longer 5-year survival rate (B).

**Figure 7:**
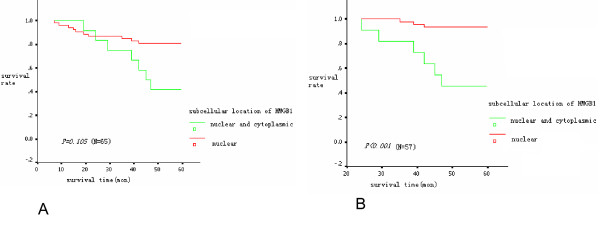
**The association between overall survival and the subcellular localization of HMGB1 in stage IIIB colon cancers**. No significant correlation with 5-year survival for patients with nuclear or nuclear and cytoplasmic expression of HMGB1 in cancer cells was observed because the survival curves crossed at 24 months (A), nuclear and cytoplasmic expression of HMGB1 in cancer cells was inversely associated with prognosis after 24 months of follow-up (B).

By the end of the 5-year follow-up period, 52 patients were alive, making the 5-year survival rate equal to 72% in this group of patients. Kaplan-Meier survival analysis indicated that a higher density of either CD3+ or CD45RO+ cells within the tumor was associated with a higher 5-year survival rate (Fig. 6). However, the association between the patterns of HMGB1 expression and 5-year survival rate was complicated. The survival curves crossed at 24 months between patients with nuclear-only expression of HMGB1 and those with co-expression of nuclear and cytoplasmic HMGB1 (Fig.7A). Kaplan-Meier survival analysis revealed no statistical difference in survival between the patients with nuclear-only expression of HMGB1 and those with co-expression of nuclear and cytoplasmic HMGB1 within 24 months after surgery. After 24 months, the patients with the co-expression of nuclear and cytoplasmic HMGB1 showed a lower 5-year survival rate compared with the patients with nuclear-only expression of HMGB1 (Table 5, Fig. 7B).

**Table 5:** Relationship between the localization of HMGB1 and prognosis after 24 months of follow-up (N = 57)

	Subcellular location of HMGB1		*P *value
	nuclear only	nuclear and cytoplasmic	
Survival time (months)			< 0.001
≥60	43	5	
≥24and < 60	3	6	

### Relationship between the density of TILs, the level or subcellular location of HMGB1, clinical characteristics, and 5-year survival rate as analyzed via multivariate survival analysis

The Cox regression model revealed that only those patients with a higher density of CD45RO+ cells within the tumor stroma had a longer survival, whereas no relationship was observed between the survival and the density of CD3+ cells. There was also no relationship between the survival and the levels or the subcellular distribution (nucleus only or nucleus combined with cytoplasm) of HMGB1 in primary tumors. These results indicated that in this group of patients, the density of CD45RO+ cells within the tumor was independently prognostic (Table 6). The same result occurred in the time-dependent multivariate survival analysis (data not shown).

**Table 6:** Multivariate survival analysis (N = 72)

	B	SE	Wald	df	**Sig**.	Exp(B)	95%CI for Lower	Upper Exp(B)
Pathological grade	.375	.257	2.120	1	.145	1.454	.878	2.408

Gender	-.111	.270	.168	1	.682	.895	.527	1.520

Age	.019	.242	.006	1	.936	1.020	.634	1.638

Tumor sites	.015	.253	.004	1	.952	1.015	.619	1.666

Density of CD3+ cells	-.569	.351	2.632	1	.105	.566	.285	1.126

Density of CD45RO+ cells	-.883	.376	5.524	1	.019	.414	.198	.864

Level of HMGB1	.099	.300	.110	1	.740	1.105	.613	1.989

Localization of HMGB1	-.130	.229	.322	1	.570	.878	.560	1.376

## Discussion

To determine the role of HMGB1 in the progression of colon cancer, this study examined the patterns of HMGB1 expression and their relationship with the infiltration of T cells and the 5-year survival rate in patients with stage IIIB colon cancer. The results showed that the density of CD45RO+ T cells within the cancer tissue was independently prognostic. More importantly, the co-expression pattern of nuclear and cytoplasmic HMGB1 in cancer cells was inversely related to the infiltration of CD3+ or CD45RO+ T cells and patient 5-year survival rate. These data indicate that the pattern of HMGB1 expression is related to the progression of colon cancer.

The role of TILs in colorectal cancer has been discussed extensively during the past 3 decades, but controversy continues. Recently, two studies have focused on the role of memory T cells in the progression of colorectal cancer. Pagès et al. [16] concentrated on the role of TILs in the early metastatic invasion of 959 cases of colorectal cancer and observed increasing levels of TILs, especially CD45RO+ memory cells, in tumors without pathologic signs of early metastatic invasion. The increasing levels of TILs were correlated with a less advanced pathologic stage and increased survival, indicating the potential of CD45RO+ memory cells for limiting early metastasis of colorectal cancer. Galon et al. [17] explored the role of the adaptive immune response in controlling growth and recurrence in 454 cases of human colorectal cancer and found that patients with a higher density of CD3+ and CD45RO+ cells in the tumor center and the invasive margins of the tumor samples had prolonged disease-free survival, indicating that the type, density, and location of immune cells within the tumor samples were predictors of survival. Both of these studies revealed the protective role of CD45RO+ memory cells in early colorectal cancer. The present study revealed that the infiltration of CD45RO+ cells was also protective in locally advanced colon cancer. As 72% of CD45RO+ cells were also positive for CD3, the protective CD45RO+ cells in colon cancer should be predominantly CD3+ CD45RO+ memory T cells. Although a higher density of CD3+ T cells was also associated with a favorable prognosis in univariate analysis, this prognostic value was not apparent in multivariate analysis, an observation that is also consistent with a recent study [39].

Since the memory T cells residing in colon cancers result from an acute immunologic response, this study further examined whether HMGB1 was involved in this process. A higher level of HMGB1 was associated with a poorer prognosis or more malignant phenotypes in multiple solid tumors, such as nasopharyngeal, lung, skin, hepatic, gastric, and prostate cancers, with the exception of breast cancer [40-46]. Previous studies have shown that the co-expression of RAGE and HMGB1 led to enhanced migration and invasion by colon cancer cell lines. Furthermore, increased RAGE expression in colon cancers has been associated with atypia, adenoma size, and metastasis to other organs. Additionally, stage I tumors have relatively lower expression levels of HMGB1, whereas stage IV tumors have near-universal expression of HMGB1 [47-52]. However, only a few studies have shown the association between HMGB1 expression and 5-year survival in colon cancer patients. Recent clinical evidence showed that the overexpression of HMGB1 in colorectal cancer was related to malignant phenotypes and shorter survival times [53-55]. To decrease the bias from factors like TNM stage and rectal cancers, this study analyzed specimens only from stage IIIB colon cancer patients. The results indicated that the co-expression of nuclear and cytoplasmic HMGB1 in colon cancer cells was associated with a poor prognosis. In normal colon mucosa, the expression of HMGB1 was observed only in the nucleus. In colon cancer cells, the co-expression pattern of HMGB1 in the nucleus and cytoplasm was observed in a subset of cancer cells, whereas nuclear-only expression of HMGB1 existed in most colon cancer cells. The survival curves of patients with the nuclear-only expression pattern and those with a pattern of co-expression crossed at 24 months after surgery, a result that might derive from the relatively small number of cases in this study, as no statistically significant difference was detected. However, Kaplan-Meier survival analysis showed that the patients with the pattern of co-expression of HMGB1 had a lower 5-year survival rate after 24 months of follow-up than those patients with HMGB1 expression in the nucleus only. This effect suggested that the pattern of co-expression of HMGB1 in cancer cells interfered with the long-term survival by partially modulating the immune response in a subset of colon cancer patients.

Because HMGB1 is a DAMP, the association between the expression of HMGB1 and the infiltration of TILs was further analyzed in this study. The results showed that co-expression of nuclear and cytoplasmic HMGB1 was also inversely related to the infiltration of CD45RO+ cells and CD3+ cells, again arguing that the effect of the pattern of co-expression of HMGB1 on survival was associated with the local modulation of the immunologic response by HMGB1. The mechanism underlying this inverse association is still elusive. One possibility is that HMGB1 secreted by colon cancers promotes the angiogenesis switch and increases cancer cell invasiveness [56-59]. Another potential mechanism is that extracellular HMGB1 triggers the inflammatory cascade, modulates the local immunologic microenvironment towards tolerance by polarizing the response of helper T cells and improperly activating macrophages and DCs [60-64]. There is evidence suggesting that both aforementioned mechanisms might contribute to the progression of colon cancer. For example, in colon cancer cell lines, E-selectin downregulated the cellular expression of HMGB1 but enhanced the release of HMGB1 into the culture medium. The released HMGB1, in turn, activated endothelial cells to express E-selectin [65]. Additionally, the higher level of HMGB1 detected in DCs existed in the metastatic lymph nodes of colon cancer patients [66]. Therefore, HMGB1-releasing colon cancer cells might promote effective angiogenesis and immune escape.

Several lines of evidence indicate that chemotherapy or radiation therapy induce the release of HMGB1 from dying cells, and this damage-related, acute release of HMGB1 is involved in the protective immune response [67-69]. This study observed that the co-expression of HMGB1 in the nuclear and cytoplasm was associated with poorer prognoses and lower infiltration of CD45RO+ cells, suggesting that spontaneous release and damage-induced release of HMGB1 might act differently in the progression of colon cancer. However, This study was just a descriptive one, more functional work should be done to reveal the mechanisms underlying this process. Therefore, different strategies should be considered when targeting HMGB1 under the conditions of spontaneous chronic release and damage-related, acute release of HMGB1 [70-72].

## Conclusion

This study observed that the co-expression of nuclear and cytoplasmic HMGB1 was inversely associated with the infiltration of CD45RO+ cells and the 5-year survival rate in patients with stage IIIB colon cancer. These observations indicated that the distribution patterns of HMGB1 contribute to the progression of colon cancer. The co-expression of nuclear and cytoplasmic HMGB1 might be used as a marker for poor survival in patients with local advanced colon cancer.

## Competing interests

The authors declare that they have no competing interests.

## Authors' contributions

WXJ, DY, ZX, PZZ, WDS, and ZLM carried out the case collection. PRQ, LCY, and YXJ carried out the immunohistochemical staining and flow cytometry analyses. ZXS and ZYX conceived the study, participated in its design and coordination, and helped draft the manuscript. All authors read and approved the final manuscript.

## Pre-publication history

The pre-publication history for this paper can be accessed here:

http://www.biomedcentral.com/1471-2407/10/496/prepub
